# Nasal immunization with H7 flagellin protects mice against hemolytic uremic syndrome secondary to *Escherichia coli* O157:H7 gastrointestinal infection

**DOI:** 10.3389/fcimb.2023.1143918

**Published:** 2023-05-16

**Authors:** Alan Mauro Bernal, Fernando Nicolás Sosa, María Florencia Todero, Daniela Romina Montagna, Mónica Elba Vermeulen, Romina Jimena Fernández-Brando, María Victoria Ramos, Agustina Juliana Errea, Martin Rumbo, Marina Sandra Palermo

**Affiliations:** ^1^ Laboratorio de Patogénesis e Inmunología de Procesos Infecciosos, Instituto de Medicina Experimental (IMEX), Consejo Nacional de Investigaciones Científicas y Técnicas (CONICET)-Academia Nacional de Medicina, Buenos Aires, Argentina; ^2^ Laboratorio de Fisiología de Procesos Inflamatorios, IMEX CONICET-Academia Nacional de Medicina, Buenos Aires, Argentina; ^3^ Laboratorio de Oncología Experimental, IMEX CONICET-Academia Nacional de Medicina, Buenos Aires, Argentina; ^4^ Laboratorio de Células Presentadoras de Antígenos y Respuesta Inflamatoria, IMEX CONICET-Academia Nacional de Medicina, Buenos Aires, Argentina; ^5^ Instituto de Estudios Inmunológicos y Fisiopatológicos - CONICET - Universidad Nacional de La Plata, La Plata, Argentina

**Keywords:** Shiga toxin-producing *Escherichia coli*, intestinal infection, HUS, flagellin, immunization, mouse model, immune-response, Shiga toxin

## Abstract

**Introduction:**

Shiga-toxin (Stx) producing *Escherichia coli* (STEC) O157:H7 is the most frequent serotype associated with hemolytic uremic syndrome (HUS) after gastrointestinal infections. Protection against HUS secondary to STEC infections has been experimentally assayed through the generation of different vaccine formulations. With focus on patients, the strategies have been mainly oriented to inhibit production of Stx or its neutralization. However, few approaches have been intended to block gastrointestinal phase of this disease, which is considered the first step in the pathogenic cascade of HUS. The aim of this work was to assay H7 flagellin as a mucosal vaccine candidate to prevent the systemic complications secondary to *E. coli* O157:H7 infections.

**Materials and methods:**

The cellular and humoral immune response after H7 nasal immunization in mice were studied by the analysis of systemic and intestinal specific antibody production, as well as cytokine production and lymphocyte proliferation against H7 flagellin ex vivo.

**Results:**

Immunized mice developed a strong and specific anti-H7 IgG and IgA response, at systemic and mucosal level, as well as a cellular Th1/Th2/Th17 response. H7 induced activation of bone marrow derived dendritic cells *in vitro* and a significant delayed-type hypersensitivity (DTH) response in immunized mice. Most relevant, immunized mice were completely protected against the challenge with an *E. coli* O157:H7 virulent strain *in vivo*, and surviving mice presented high titres of anti-H7 and Stx antibodies.

**Discussion:**

These results suggest that immunization avoids HUS outcome and allows to elicit a specific immune response against other virulence factors.

## 1 Introduction

Several serotypes of Shiga toxin (Stx) producing *E. coli* (STEC) have been associated with hemolytic uremic syndrome (HUS), a life-threatening condition that mainly affect healthy children under 5-year- old. However, Stx type 2a and/or 2c-producing *E. coli* (O157:H7), is the serotype most frequently associated with a poor outcome after gastrointestinal infection in North America, the UK and Argentina (Pianciola and Rivas, 2018; Alconcher et al., 2021; Butt et al., 2022). The main reservoir for O157:H7 is cattle and zoonotic transmission occurs after consumption of insufficiently cooked meat or deficiently pasteurized dairy products, inadequately washed vegetables and fruits, or exposure to contaminated water. Besides, O157:H7 *E. coli* can be person-to-person transmitted through orofecal route. The situation in Argentina is worrying because O157:H7 *E. coli* associated HUS occurs as sporadic cases throughout the year, together with outbreaks mainly in the warm months of the year, probably as the consequence of a high circulation of highly virulent STEC strains within the community (Carbonari et al., 2022). In addition, early detection of O157:H7 *E. coli* infections during the gastrointestinal phase is scarce (Pianciola et al., 2014; Balestracci et al., 2021). This fact prevents the application of control procedures to avoid the spread of the pathogen, and/or the early application of therapeutic strategies, although a specific treatment for HUS is not currently present and a supportive therapy is generally applied. The most efficient way to solve the problems associated with infectious diseases is to develop preventive strategies such as an appropriate vaccination. In this regard, different alternatives have been proposed for preventing HUS depending on the target chosen, i.e., reservoir or patients. At present, there is not any approved vaccine for human use, but a number of drugs have been experimentally assayed to prevent systemic consequences of STEC infections. Since the major pathogenic factor of STEC is Stx, strategies were mainly oriented to inhibit Stx production and release, or to neutralize Stx in blood circulation (Mejias et al., 2016; Hiriart et al., 2018; Goldstein et al., 2021). However, few approaches have been intended to block the gastrointestinal phase of this disease, which is considered the first step in the pathogenic cascade of HUS. Several pieces of evidence support that protection against non-invasive enteric infections such as cholera and enterotoxigenic *Escherichia coli* (ETEC) diarrhea is mainly mediated through antigen-specific secretory IgA (SIgA) antibodies produced locally in the mucosa (Czerkinsky and Holmgren, 2015). Similarly, we recently demonstrated that protection against HUS associated with STEC-infection in mice is dependent on the B cell response and IgA specific antibody-response in the intestine (Bernal et al., 2021).

Thus, a novel alternative to prevent HUS secondary to STEC infections may be a mucosal vaccination to induce a robust humoral immune response in the intestine. In this regard, it has been shown that nasal vaccination stimulates mucosal and systemic humoral and cellular responses against infectious agents (Olivera et al., 2014). Flagellin is the structural protein subunit of the flagellum and is a promising immunogen to be used by the mucosal route because it acts as pathogen-associated molecular patterns (PAMPs). Flagella from O157:H7 *E. coli* are not only the motility apparatus of bacteria but also participate in adherence of the bacteria to the host cells (Giron et al., 2002). Besides, it has been identified as a major virulence factor in the intestine due to a direct interaction with the mucus layer, *via* Muc2 (Mahajan et al, 2009; Best et al., 2005; McWilliams and Torres, 2014). Purified flagella were capable of interacting with intestinal epithelial cells *in vitro* and of interfering with bacterial binding. Finally, it was shown that the expression of STEC flagella is downregulated after contact with the epithelium, suggesting that it may be required only for early stages of binding and provides a means for the type-three secretion system (T3SS) to increase transcription of its components and mediate a long-term binding event *via* the A/E lesion (Mahajan et al, 2009). Other authors have proposed that addition of H7 flagellin to a T3SS-based subunit vaccine (EspA and Intimin alone) could increase the effectiveness of vaccine formulations for cattle (McNeilly et al., 2015).

Flagellin triggers a strong and sustained antibody response, even in the absence of any adjuvant. Flagellin is the ligand for toll like receptor five (TLR5) (Hayashi et al., 2001), and is a potent systemic and mucosal adjuvant (Bates et al., 2008), which is probably dependent on its ability to induce maturation of dendritic cells (DC) (Agrawal et al., 2003). In this regard, flagellin has been reported to typically elicit Th2-type immune responses (Didierlaurent et al., 2004). However, Th1 responses in mice (McSorley et al., 2002) and the secretion of IL-12 by human DC (Means et al., 2003) after treatment with flagellin were also reported. Considering this background, the aim of this work was to immunize mice with H7 flagellin to prevent systemic sequelae secondary to gastrointestinal O157:H7 *E. coli* infections. We analyzed H7-specific humoral and cellular immune responses, at both systemic and intestinal level in H7-immunized mice. Furthermore, immunized and control mice were challenged with O157:H7 *E. coli*. Survival rates and kidney damage were evaluated as an *in vivo* protection test about the efficacy of H7-vaccination.

## 2 Materials and methods

### 2.1 Bacterial strains

The enterohemorrhagic *Escherichia coli* strain used in this study for mice infection was isolated from fecal specimens of a patient with HUS (125/99) (Amigo et al., 2015). This strain belonged to the serotype O157:H7, and harbored the *eae*, *ehxA* and *stx*_2a_ genes; but not *stx*_1_ (Brando et al., 2008). This strain was transformed with a plasmid carrying only ampicillin resistance (125/99 pWSK29) (Fernández-Brando et al., 2020).

For obtaining H7 flagellin, we used an isogenic strain of 125/99 in which the *stx2* gene was deleted (ΔStx2) (Albanese et al., 2015) to avoid the presence of Stx2 in bacterial supernatants.

### 2.2 Bacterial growth

Briefly, single colonies of 125/99 ΔStx2 (ΔStx2) or 125/99 pWSK29 (125/99 pW) picked up from LB agar plates were grown in 10 mL of tryptic soy broth (TSB) (Difco, Le Point de Claix, France) in a 37°C shaker until the culture reached the exponential phase. Then, a dilution 1/100 of this culture was made into an Erlenmeyer flask containing TSB and grown overnight at 37°C without shaking. The culture was centrifuged for 30 min at 2000 rpm, bacterial pellets were washed twice and resuspended in phosphate-buffered saline (PBS). The 125/99 pW cultures were carried on in the presence of 100 µg/mL ampicillin.

### 2.3 Bacterial challenge

A calibration curve was made plotting the number of 125/99 pW CFU/mL vs optical density at 600 nm (OD_600_). For mice challenge, the 125/99 pW pellet was washed twice and resuspended in PBS to obtain a bacterial dose ranging from 1.0 x10^6^ to 2.5 x10^6^ CFU/mL by using the calibration curve. The number of CFU was obtained by plating dilutions on LB agar plates in order to confirm inocula.

### 2.4 Flagellin purification

Flagellin was prepared as previously described (Hiriart et al., 2012; Kreutzberger et al., 2022). Briefly, H7 flagellin was obtained by shearing flagella from ΔStx2 bacterial surface by vortexing for 5 min at 30 Hz. After centrifugation at 13000 rpm for 10 min, the bacteria-free supernatant containing the sheared flagella was concentrated by ultracentrifugation (165000 g for 1.5 h at 4°C in a Beckman ultracentrifuge with a 70Ti rotor, Fullerton, CA, USA). The flagella pellet was resuspended in PBS and heated for 10 min at 80°C to release monomers (1-5 mg/L culture). The final suspension was centrifuged at 13000 rpm for 15 min at 4°C to precipitate bacterial debris. Protein concentration was determined by Bradford microassay (Bio-Rad, Hercules, CA, USA) and flagellin activity was evaluated in a reporter cell line Caco-2 CCL20:luc (Hiriart et al., 2012; Iraporda et al., 2016). Flagellin from *Salmonella enterica serovar typhimurium* ATCC14028 (FliC) was kindly provided by Dr Rumbo (Hiriart et al., 2012). Briefly, the cells were grown in Dulbecco’s Modified Eagle’s Minimum Essential Medium (DMEM; Gibco BRL Life Technologies, Rockville, MD, USA) supplemented with 15% heat-inactivated fetal-bovine serum (FBS; Natocor, Córdoba, Argentina), antibiotics (100 U/mL penicillin/streptomycin) (EMEVE Microvet SRL Laboratories, Buenos Aires, Argentina) and 2 mM L-glutamine (EMEVE). Caco-2-CCL20:luc cells were used after 8 days of culture. Confluent Caco-2-CCL20:luc cells cultured in 48-well plates were stimulated with H7 flagellin (1 µg/mL) or FliC (1 µg/mL), as a positive control, during 6 h at 37°C under 5% CO_2_ in a humidified atmosphere. Cells without any treatment was included as a negative control lacking stimulation. After incubation, cells were lysed with lysis buffer (Promega, Madison, WI, USA), and luciferase activity was assessed using the Luciferase Assay Kit (Promega, Madison, WI, USA) following manufacturer’s instructions and measured in a luminometer (Luminoskan TL Plus) to determine arbitrary units of luminescence (AUL). Purity of H7 flagellin was verified by 12% sodium dodecyl sulfate-polyacrylamide gel electrophoresis (SDS-PAGE) followed by Coomassie Blue R250 (Sigma, St Louis, MO, USA) or silver staining; single bands of the anticipated molecular weight for H7 flagellin were identified (MW: 70 kDa) by both methods. Contaminating endotoxin levels (<60 pg LPS/µg H7 flagellin) were determined by Limulus amebocyte lysate test (LAL QCL-1000) (Lonza, Walkersville, MD, USA) using lipopolysaccharide (LPS) from *E. coli* O111:B4 as standard (Sigma). Although bacterial growth and culture conditions used were not the optimal to express (T3SS), we cannot rule out the contamination with traces of these proteins.

### 2.5 Western blotting (WB)

H7 flagellin was separated through a 12% SDS-PAGE as mentioned above and transferred onto Hybond ECL nitrocellulose membrane (GE Healthcare Life Sciences, Marlborough, MA, USA) using a Trans-Blot electrophoretic transfer cell (Bio-Rad). After membrane blocking with 5% (w/v) milk powder (Sigma) in PBS at 4°C overnight, it was sequentially incubated first with 1/2000 rabbit polyclonal anti-H7 flagellin (Abcam, Boston, MA, USA), 1/50000 plasma from H7-immunized BALB/c mice or 1/10000 plasma from control BALB/c mice, and second with 1/3000 HRP-conjugated goat polyclonal anti-rabbit IgG or anti-mouse IgG (Invitrogen, San Diego, CA, USA), all diluted in PBS containing 5% (w/v) milk powder. Incubations were carried out at room temperature for at least 1 h on a platform shaker and were washed 3 times during 5 min with 0.1% (v/v) Tween20 in PBS before and after each antibody step. Specific bands were visualized by enhanced chemiluminescence (ECL) method (GE Healthcare Life Sciences).

### 2.6 Mice

BALB/c mice were purchased from the Charles River Laboratory and maintained in specific pathogen-free (SPF) conditions at the animal facility of the IMEX-CONICET-Academia Nacional de Medicina, Buenos Aires, Argentina. Mice were housed in standard polypropylene transparent cages under environmentally controlled conditions (Temperature, 24 ± 2°C; Humidity, 50 ± 10%) with a 12 h light: 12 h dark cycle.

### 2.7 Ethics statement

The Institutional Animal Care and Use Committee at IMEX-CONICET-Academia Nacional de Medicina approved all procedures in accordance with the principles set forth in the Guide for the Care and Use of Laboratory Animals (National Research Council (US) Committee, 2011), (protocol number 86/2021). Health and behavior of mice were assessed twice a day. Any unnecessary pain, discomfort or injury to animals was avoided. Mice becoming moribund (with a weight loss greater than 30% of its initial value and/or plasma BUN levels higher than 100 mg%) were humanely euthanized by intraperitoneal administration of ketamine (75 mg/kg mouse) and xylazine (15 mg/kg mouse) with subsequent cervical dislocation. Institutional Animal Care & Use Committee (IACUC) guidelines were used to define humane endpoints.

### 2.8 Mouse models

#### 2.8.1 Immunization scheme

Six to 8 weeks old BALB/c mice (males and females indistinctly) (n=4-5 per treatment, per experiment) were intranasally immunized with 20 µl of a purified H7 flagellin stock of 500 µg/mL PBS (10 µg of purified H7 flagellin/mouse). Mice received 3 doses, without adjuvant, at 10-day intervals. An additional control group (n=4) was immunized with FliC (10 µg/dose/mouse) following the same scheme. Blood and stools samples were obtained at different times post immunization (on day 17, 27, 41, 62 and 83 post first dose). Blood samples (50 µL) were obtained by submandibular puncture with a hypodermic needle (25Gx5/8”) and collected into a tube with 5 µL of 100 mM ethylenediaminetetraacetic acid (EDTA). Blood was centrifuged at 4000 rpm for 5 min to separate plasma which was stored at -20°C until IgG anti-H7 determination by enzyme-linked immunosorbent assay (ELISA). Stools were weighed and adjusted to 250 mg/mL in PBS with 1 mM phenylmethylsulfonylfluoride (PMSF) (Sigma). After vigorous homogenization with vortex, feces were centrifuged (13300 rpm for 10 min) and supernatants were stored at -80°C until anti-H7 IgA and IgG determination by ELISA. Blood and feces from FliC immunized control group was similarly tested for anti-H7 and anti-FliC IgG and IgA antibodies.

#### 2.8.2 125/99 pW infection challenge

Non-immunized, FliC- or H7-immunized mice (on day 41 post first dose) (n=4-6 per treatment, per experiment) were treated 6 hours before infection and 18 hours post infection (p.i.) with ampicillin (1 mg/mouse) by oral gavage using a stainless-steel cannula (model 7.7.1; 0,38 mm x 22G, Harvard Apparatus, USA) as previously described (Zangari et al., 2013). For infection, mice were inoculated with a dose of 125/99 pW strain (0.1 mL of a bacterial suspension containing 1.0-2.5 x10^6^ CFU/mL). This dose (1.0-2.5 x10^5^ CFU/mouse) leads mice to death at day 7 or 8 p.i. (1LD). Mice were monitored and weighted all subsequent days until the end of experiments, when survivors were euthanized. Blood samples were taken at 7- and 15-days p.i. for laboratory analyses, which included anti-H7 IgG and anti-Stx2B IgG determination, total and differential blood cell counts in an automated hematologic counter (Abacus Junior Vet, Diatron, USA) and determination of urea in plasma by a commercial kit (Urea Color Kit, Wiener Lab, Rosario, Argentina) following the manufacturer’s instructions. Blood urea nitrogen (BUN) levels were expressed as mg%. Stools were also collected to determine anti-H7 IgG and IgA as described below at day 7 and 15 p.i.

#### 2.8.3 Bacterial colonization

Control and FliC or H7-immunized mice were euthanized at day 6 p.i. with 125/99 pW strain to determine intestinal colonization as previously described (Bernal et al., 2021). Briefly, 5 cm of the small and large intestine and the cecum were excised. After removal of the feces, tissues were homogenized separately in 0.5 mL PBS. CFU were determined by plating dilutions of the homogenized intestinal tissues onto MacConkey agar plates. After incubation for 16 h at 37°C, non-sorbitol-fermenting colonies were identified and counted. The number of CFU per intestinal segment was calculated by considering the CFU per milliliter and the total volume of each homogenized tissue.

#### 2.8.4 Gut permeability assay

To measure the intestinal barrier function, a fluorescein isothiocyanate (FITC)- conjugated dextran (FITC-Dx; mean molecular weight 4 kDa) (Sigma-Aldrich, Oakville, ON, USA) assay was carried out as previously described (Bernal et al., 2021). Briefly, 0.1 mL PBS containing 80 g/L FITC-Dx was orally administered at day 6 post 125/99 pW infection. The fluorescence of FITC-Dx was measured in plasma after 4 h of treatment. Blood samples were collected as mentioned above, and the plasma was diluted in a 1:1 ratio (100 uL final volume) with 15mM carbonate- 25mM bicarbonate buffer, pH 9.6. Fluorescein fluorescence was determined with a microvolume fluorimeter (Thermo Scientific NanoDrop, USA) using an excitation wavelength of 495 nm and a detection wavelength of 514 nm.

#### 2.8.5 Delayed type hypersensitivity reaction (DTH)

Control and H7-immunized mice (n=3-4 per treatment, per experiment) were intradermally inoculated with 5 µg of purified H7 in 0.04 mL PBS in the right footpad, while the same volume of PBS was injected in the left, on day 83 post first dose. The DTH reaction was evaluated by measuring the thickness of each footpad using a caliper (Oditest, H. C. Kröplin, Schlüchtern, Germany) at 48 and 72 h post inoculation. DTH reaction was expressed as Δ swelling, calculated as the difference in swelling between both footpads at each time (Δmm swelling= mm right footpad – mm left footpad).

### 2.9 Assessment of humoral response

#### 2.9.1 Determination of anti-flagellin IgA and IgG in feces supernatants and plasma by ELISA

Ninety-six well MaxiSorp plates (Greiner Bio-One GmbH, Frickenhausen, Germany) were coated with 0.25 µg of purified H7 or FliC/well in PBS (pH 7.4) overnight at 4°C. Wells were washed with 0.05% Tween20 in PBS (PBS-T), blocked with 3% milk powder in PBS (blocking buffer) for 1.5 h at room temperature, washed again with PBS-T and finally incubated with plasma or feces supernatants 1/2 serial dilutions overnight at 4°C. Then, wells were washed with PBS-T and incubated with 1/3000 HRP-conjugated goat anti-mouse IgA (Invitrogen) or HRP-conjugated goat anti-mouse IgG (Invitrogen) diluted in blocking buffer for 1.5 h at room temperature with orbital agitation. (2 mg/mL o-phenylenediamine (OPD) (Sigma) and 0.3% H_2_O_2_ in 0.1M citrate- 0.2M phosphate buffer, pH 5.0) and incubated for 10 min at room temperature in darkness. The reaction was stopped with 2M H_2_SO_4_ and absorbance at 492 nm was measured on Asys UVM340 (microtiter plate reader Biochrom Ltd., Cambridge, UK). Results were expressed as OD at 492 nm (OD_492_) units which represent the value obtained for each sample minus the OD_492_ obtained for its non-specific binding.

#### 2.9.2 Determination of anti-Stx2 IgG in plasma by ELISA

Stx2B-specific IgG in plasma was analyzed by ELISA as previously described (Mejias et al., 2013). Briefly, 96-well MaxiSorp plates (Greiner Bio-One) were coated with 5 μg of purified Stx2B subunit conjugated to histidine per mL of 15 mM carbonate-25 mM bicarbonate buffer (pH 9.6) overnight at 4°C. Wells were washed with PBS-T and blocked with 2% bovine serum albumin (BSA) (Sigma) in PBS (blocking buffer) for 1.5 h at room temperature. After washing with PBS-T, the plates were incubated overnight at 4°C with 1/10 diluted mouse plasma. Wells were washed with PBS-T and the plates were incubated with 1/3000 HRP-conjugated goat anti-mouse IgG (Invitrogen) diluted in blocking buffer for 1.5 h at room temperature with orbital agitation. Then, the chromogenic substrate was added as described above. Results were expressed as OD_492_, which represents the value obtained for each sample minus the OD_492_ obtained for its non-specific binding. Plasma from not immunized nor infected BALB/c mice was used as negative control, while serum from BLS-Stx2 immunized BALB/c mice (Mejias et al., 2013) was used as positive control.

### 2.10 Bone marrow dendritic cells culture (BMDC)

Bone marrow was harvested from the femurs and tibias of BALB/c mice. After lysing red blood cells with ammonium chloride buffer (0.15M NH_4_Cl, 10mM NaHCO_3_ and 0.1mM EDTA, pH 7.4), BM cells were plated in 90 mm cell culture dish (Jet Biofil, Guangzhou, China) at a density of 1 x10^6^ cells/mL. Cells were cultured with recombinant mouse GM-CSF (10 ng/mL) and IL-4 (5 ng/mL) (Peprotech, Cranbury, NJ, USA) in RPMI 1640 medium (Gibco, Invitrogen, San Diego, CA, USA) supplemented with 10% heat-inactivated fetal bovine serum (FBS) (Natocor), Córdoba, Argentina, antibiotics (100 U/mL penicillin/streptomycin) (EMEVE), 0.05 mM β-mercaptoethanol (Sigma), and 2 mM L-glutamine (EMEVE) (complete RPMI 1640 medium) for 7 days with media changes on days 2, 4 and 6 at 37°C under 5% CO_2_ in a humidified atmosphere. On day 7, BMDC were harvested, counted, and replated in a 96-well cell culture plate at 5 x10^4^ cells/well in 100 uL complete RPMI 1640 medium. Cells were stimulated with purified H7 at 1 µg/mL (200 ng/well), or LPS from *E. coli* O111:B4 at 1 µg/mL (200 ng/well) (Sigma). Twenty-four hours later, supernatants were harvested for cytokine analysis and cells were collected for flow cytometry analysis. BMDC were incubated for 30 min at 4°C sheltered from the light with antibodies against the following clusters of differentiation (CD) which allow to identify dendritic cells (CD11c) and mature dendritic cells (CD86). The antibodies used were PE- conjugated rat anti-mouse CD86- and PE-Cy7- conjugated rat anti-mouse CD11c (BD Biosciences, Franklin Lakes, NJ, USA). Samples were acquired on a CyFlow Space cytometer (Sysmex Deutschland GmbH, Norderstedt, Germany). BMDC incubated without antibodies were used as controls to set the cytometer settings. BMDC labeled only with PE or PE-Cy7 were used to correct the spectral overlay (compensation tubes). BMDC were delimited by size (FSC) and granularity (SSC) in a two-parameter plot and 20000 events were acquired for subsequent analysis using FlowJo 10.0 software. Singlets were selected by exclusion by FSC-W vs FSC-A and SSC-W vs SSC-A analysis, and on the whole DC (CD11c+ events), the percentage of mature DC was determined as % of CD86 positive events.

### 2.11 Leukocyte proliferation assay

Leukocytes were obtained from lung-draining lymph nodes (LN) and spleens from control and H7-immunized mice (n=3-5 per treatment, per experiment) and labeled with 5(6)-carboxyfluorescein diacetate N-succinimidyl ester (CFSE) (Sigma). CFSE labeling was performed by incubating 5.0 x10^6^ cells/mL serum-free RPMI 1640 containing 5 µM CFSE for 10 min at 37°C. After incubation, cells were washed twice with PBS (1200 rpm 10 min at 4°C) and left at rest for 10 minutes at 37°C. Then, cells were washed twice again and single cell suspensions were plated onto 96-well cell culture plate (5 x10^5^/well) in complete RPMI 1640 medium. These cells were cultured with BMDC (5 x10^4^/well) previously stimulated with 200 ng H7 (24 hours before), or with unstimulated BMDC (control), at 37°C under 5% CO_2_ in a humidified atmosphere. After 5 days of culture, supernatants were collected for cytokine analysis and cells were incubated for 30 min at 4°C sheltered from the light with antibodies against the following CD: CD8 (to identify cytotoxic T lymphocytes); CD4 (to identify Helper T lymphocytes) or B220 (to identify B Lymphocytes). The antibodies used were: PE-conjugated rat anti-mouse CD8, PE-Cy5- conjugated rat anti-mouse CD4 and PE-Cy7- conjugated rat anti-mouse B220 (BD Biosciences). Samples were acquired on a CyFlow Space cytometer. Lymphocytes incubated without antibodies were used as controls to set the cytometer settings. Lymphocytes stained only with CFSE, or with each one of the antibodies (labelled with PE, PE-Cy5 or PE-Cy7) were used as compensation tubes. Fluorescence minus one (FMO) tubes were used to identify and gate positive populations in multicolor experiments. Lymphocytes were delimited by size (FSC) and granularity (SSC) and 20000 events were acquired for subsequent analysis using FlowJo 10.0 software. Singlets were selected by exclusion by FSC-W vs FSC-A and SSC-W vs SSC-A analysis. Lymphocyte proliferation was measured after culture as the decrease in CFSE geometric mean fluorescence intensity (MFI) compared with CFSE MFI at time 0 (no proliferation). The percentage of proliferating CD4, CD8 and B220 cells were calculated as the percentage of each population among the cells with low CFSE staining.

#### 2.11.1 Measurement of cytokines in supernatants

Concentrations of murine IFN- γ (15-2000 pg/mL), TNF-α (8-1000 pg/mL), IL-4 (4-500 pg/mL), IL-5 (4-500 pg/mL), IL-6 (4-500 pg/mL), IL-12p70 (15-2000 pg/mL) and IL-17A (4-500 pg/mL) were measured in the supernatants mentioned above using a standardized sandwich ELISA according to the manufacturer’s instructions (Invitrogen, Carlsbad, CA, USA). The sensitivity and assay range for each ELISA kit provided by the supplier is indicated in brackets. The OD was measured at 450nm (OD_450_) in a microtiter plate reader Asys UVM340. The concentration of each cytokine was calculated by extrapolating the OD_450_ obtained in the standard curve and multiplying by the dilution factor performed.

### 2.12 Statistical analysis

The sample size reported in each group was the same number used in the statistical analysis because there were not any exclusions. Data is expressed as the mean± SEM when the assumption of Gaussian distribution (Shapiro-Wilks’s test) and heteroscedasticity (Levene’s test) were confirmed and the Student’s T test or the unpaired one- or two-way analysis of variance (ANOVA) followed by a Tukey’s post-test were used according to the number of experimental groups. All data was analyzed by Prism 8.0 software (GraphPad, San Diego, CA, United States) and InfoStat software (Córdoba, Argentina), and graphically represented by Prism 8.0 software. Survival data was analyzed for significance by using Log-Rank test. The test results were considered significantly when p<0.05.

## 3 Results

### 3.1 Validation of the identity of purified flagellin

**Figure 1:**
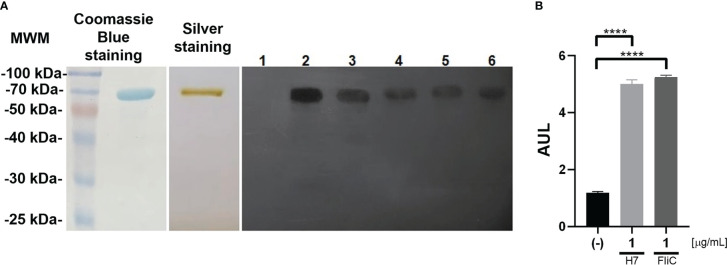
Evaluation of purified H7 flagellin: **(A)** Left panel: Coomassie blue stained SDS-PAGE of purified H7 flagellin. Central panel: Silver stained SDS-PAGE of purified H7 flagellin. Right panel: WB of purified H7 incubated with plasma from control non-immunized mice (lane 1), rabbit polyclonal anti-flagellin antibody (lane 2), or plasma from H7-immunized mice (lane 3-6). Each one of these lanes correspond to the plasma of a randomly selected mouse from each immunization scheme carried out. **(B)** Reporter cells were stimulated with purified H7 flagellin (1 µg/mL), FliC (1 µg/mL), or without any treatment (-). Results are expressed as arbitrary units of luminescence (AUL) and the mean value ± the standard error of the mean (SEM) from independent triplicates. Data was analyzed by one-way ANOVA test with Tukey’s post-test. ****p<0.0001.

The identity of H7 flagellin was verified by molecular methods as well as functional assays by determining its biological activity. A single protein of approximately 70 kDa was observed by SDS-PAGE and silver staining which reacted strongly with commercial anti-H7 antibodies following WB (**Figure 1A**). WB was performed on randomly selected plasma samples from non-immunized and one immunized mouse from each independent experiment (n=4), confirming that immunoreactivity was primarily directed against H7. Plasma from non-immunized mice did not react with purified protein.

H7 flagellin preparation was characterized in a TLR5-dependent bioactive assay using a Caco2 luciferase reporter cell line as reported previously (Hiriart et al., 2012; Iraporda et al., 2016). It was observed that 1 µg/mL of H7 elicited a strong response in this *in vitro* system, similar to the response triggered by FliC, flagellin from Salmonella *typhimurium*, used as a positive control (**Figure 1B**).

### 3.2 Humoral response after immunization

Anti-H7 antibody response was measured in fecal supernatants and plasma from control and immunized BALB/c mice by ELISA at different time points after nasal immunization.

**Figure 2:**
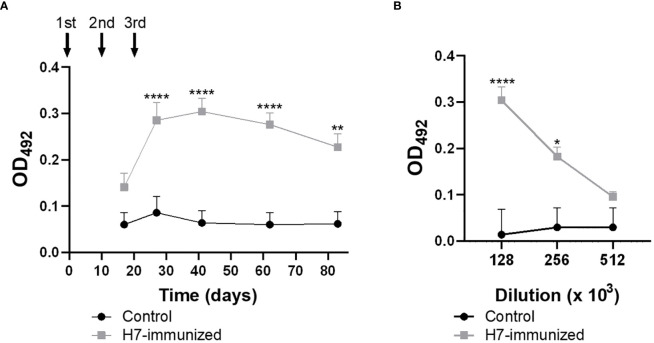
Levels of H7-specific IgG measured by ELISA in plasma. **(A)** The solid arrows indicate the timing of immunizations with H7 flagellin. Each point shows anti-H7 antibody level in plasma at 1/128000 dilution expressed as OD_492_ at each time point. **(B)** Antibody titre at the peak of maximum response post immunization (day 41 p.i.). Each point represents the mean ± SEM of 5 control and immunized mice. This experiment is representative of four biological replicates. Data was analyzed by two-way ANOVA test with Tukey’s post-test. *p<0.05, **p<0.01, ****p<0.0001.

**Figure 3:**
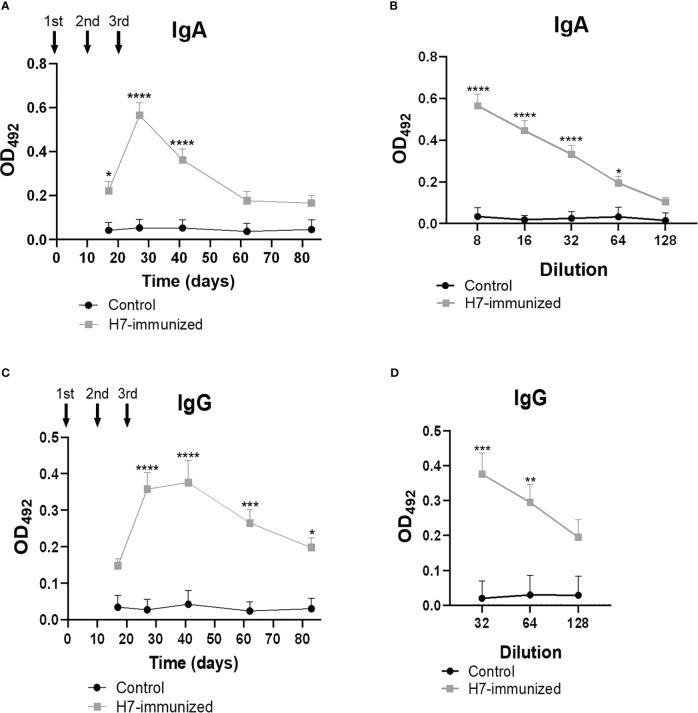
Levels of H7-specific IgG and IgA measured by ELISA in feces. The solid arrows indicate the timing of immunizations with H7 flagellin. **(A, C)** Each point shows anti-H7 IgA and IgG levels in fecal samples at 1/8 and 1/32 dilution, respectively, expressed as OD_492_ at each time point. Anti-H7 IgA **(B)** and IgG **(D)** titres at the peak of maximum response post immunization. Each point shows the mean ± SEM of 5 control and immunized mice. This experiment is representative of four biological replicates. Data was analyzed by two-way ANOVA test with Tukey’s post-test. *p<0.05, **p<0.01, ***p<0.001, ****p<0.0001.

High titres of anti-H7 IgG were detected in plasma from immunized mice (**Figures 2A, B**), which reached maximal levels after 41 days from the first immunization dose (titre 1/256000). As expected, no anti-H7 antibodies were detected in plasma from control mice. To evaluate the mucosal response, IgA and IgG specific antibodies against H7 were evaluated in fecal supernatants from both experimental groups. Anti-H7 IgA and IgG antibodies were induced in intestinal mucosa following immunization. IgA antibody levels reached a peak at 27 days post first immunization dose (titre 1/64), whereas IgG antibody levels did at 41 days (titre 1/64) (**Figures 3A–D**), in concordance with the IgG peak in plasma. The titres of both immunoglobulins were significantly different compared to the control group, which did not show specific anti-H7 antibodies. An additional control group, consisting of mice similarly immunized with unrelated flagella such as FliC, was carried out. Anti-FliC IgG antibodies in plasma (**Supplementary Figure 1A**) and anti-FliC IgA and IgG antibodies in the feces supernatants (**Supplementary Figures 2A, B**) were detected in FliC immunized mice with similar titres and kinetic to anti-H7 antibodies obtained in the H7 immunized group. However, anti-H7 IgG antibodies in plasma (**Supplementary Figure 1B**) and feces supernatants (**Supplementary Figures 2C, D**) were not significantly different between FliC-immunized and non-immunized mice. These results confirm the specificity of humoral immune response against H7 and FliC, respectively.

### 3.3 Cellular response after immunization

#### 3.3.1 Analysis of lymphocyte proliferation

**Figure 4:**
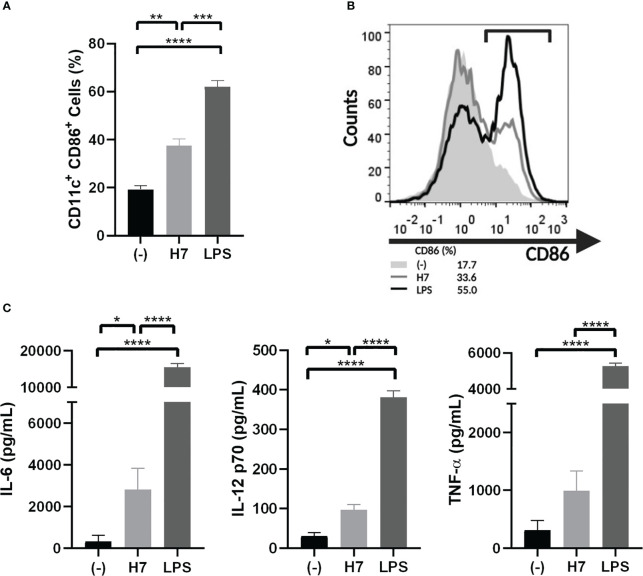
Activation of BMDC by H7 flagellin. BMDC were harvested on day 7 of culture with GM-CSF and IL-4 and stimulated for 24 h with H7 (1 µg/mL), LPS (serotype O111:B4; 1 µg/mL), or medium alone (-). **(A)** Percentage of CD11c^+^/CD86^+^ DC was assessed by flow cytometry. **(B)** Representative histograms showing the pattern of CD86 expression on DC stimulated with purified H7 (grey line), LPS (black line) or medium alone (light grey-filled histogram). **(C)** IL-6, IL-12p70 and TNF-α released in supernatants was measured by ELISA. Each bar represents the mean ± SEM of three independent experiments. Data was analyzed by one-way ANOVA test with Tukey’s post-test. *p<0.05, **p<0.01, ***p<0.001, ****p<0.0001.

In order to perform specific lymphocyte proliferation assay against H7 flagellin, BMDC from naïve mice were treated *in vitro* with 1 µg/mL H7. After 24 h of culture, BMDC activation was evaluated on CD11c^+^ cells by CD86 membrane expression and IL-6, TNF-α and IL-12p70 production in supernatants. As shown in **Figure 4** a significantly increased percentage of CD11c/CD86 positive cells and production of IL-6 and IL-12p70 was observed in BMDC cultures in the presence of H7 compared to medium alone. In contrast, no significant TNF-α secretion was detected after H7 stimulation. For all parameters, a significantly lower response was obtained after H7 stimulation in comparison to BMDC stimulated with LPS *in vitro* (**Figure 4**). These results are consistent with the previously reported flagellin TLR5-activation of murine BMDC (Hayashi et al., 2001; Bates et al., 2008).

**Figure 5:**
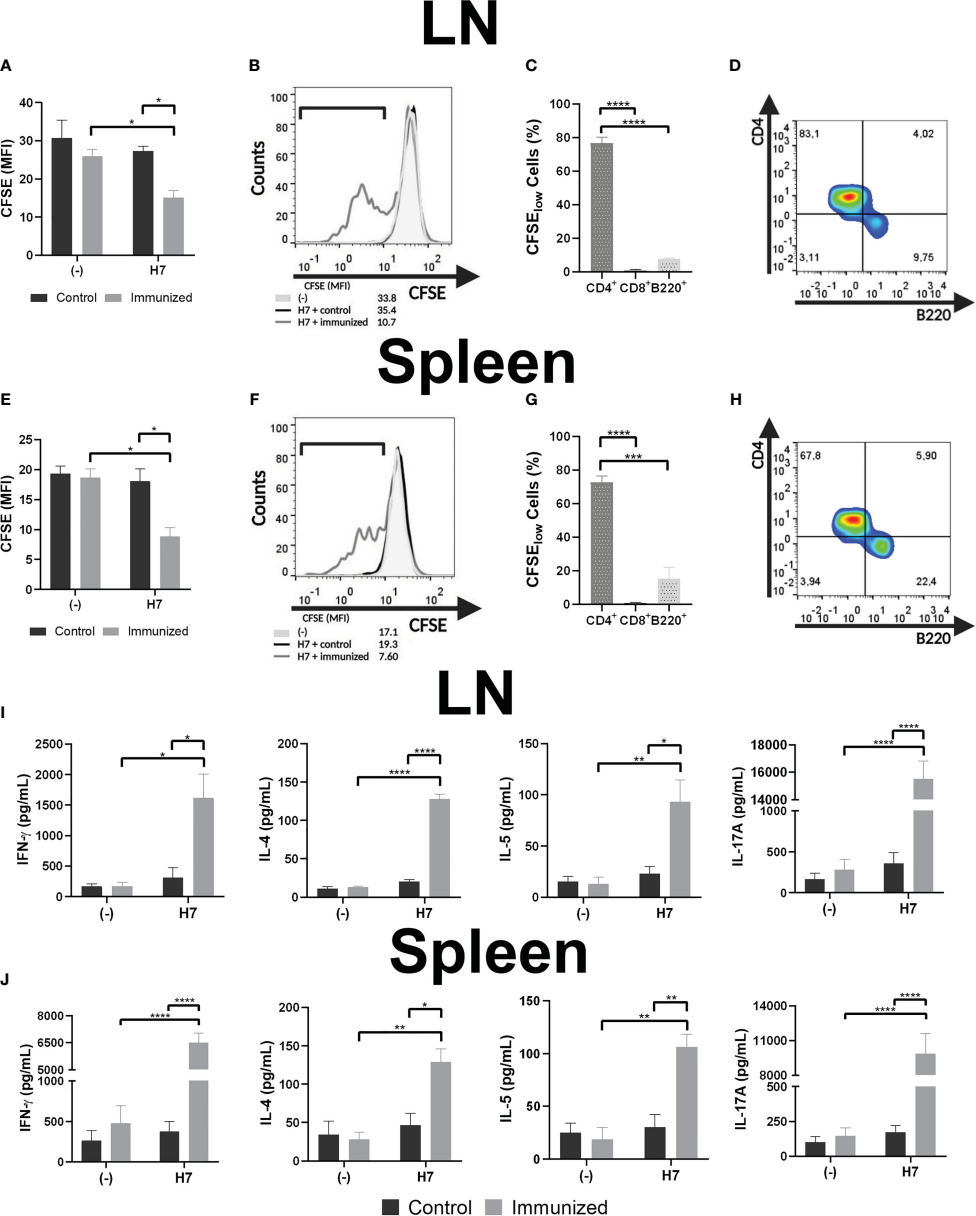
Specific lymphocyte proliferation. Cellular response was assayed *in vitro* by CFSE-stained leukocyte proliferation when leukocytes from LN **(A)** or splenocytes **(E)** from control or immunized mice (day 48 post first dose) were incubated with H7-stimulated BMDC for 5 days. Unstimulated BMDC (-) were used as negative controls. Leukocyte proliferation was determined on day 5 of culture as the decrease in CFSE MFI. **(B, F)** Representative histograms of leukocytes from an immunized mouse (grey line), or from a control mouse (black line) incubated with H7-stimulated BMDC, or leukocytes incubated with unstimulated BMDC (light grey-filled histogram) are also shown. **(C, G)** LN leukocytes and splenocytes were collected and incubated with anti-CD8, -CD4 and -B220 to determine which subsets of lymphocytes were proliferating (among low CFSE-stained cells from immunized mice) after incubation with H7-stimulated BMDC. **(D, H)** CD4 vs B220 representative dot-plots showing the major proliferating lymphocyte subsets after the above-mentioned analysis. **(I, J)** Culture supernatants were harvested to assess IFN-γ, IL-4, IL-5 and IL-17A concentrations by ELISA. Each bar shows the mean ± SEM of 4 control or 4 immunized mice. This experiment is representative of three biological replicates. Data was analyzed by two-way ANOVA test with Tukey’s post-test. *p<0.05, **p<0.01, ***p<0.001, ****p<0.0001.

Then, leukocyte suspensions were prepared from spleens and LN from control and immunized mice on the 48th day after starting the immunization. Specific leukocyte proliferation assays against H7-stimulated BMDC were evaluated by staining leukocytes with CFSE and subsequent analysis by flow cytometry after 5 days of co-culture. **Figures 5A, B, E, F** show that the CFSE MFI of LN and spleen cellular suspensions from immunized mice were significantly lower than the corresponding MFI from control mice. In the same line of evidence, a higher percentage of cells in LN and spleen cultures from immunized mice showed a lower CFSE MFI compared to control mice (LN: H7 + immunized= 30.1 ± 11.0%*; (-) + immunized= 9.0 ± 5.1%*; H7 + control= 8.9 ± 6.2%*; (-) + control= 4.7 ± 4.6%; Spleens: H7 + immunized= 40.4 ± 5.4%#; (-) + immunized= 8.6 ± 2.9%#; H7 + control= 10.9 ± 3.6%#; (-) + control= 6.1 ± 2.1%; n=3; * p<0.05; #p<0.001). In parallel, lymphocytes from LN and spleen were collected and stained with anti-CD8, -CD4 and -B220 to determine which subset of lymphocytes were proliferating after incubation with H7-stimulated BMDC. We found that CD4^+^ lymphocytes were the major proliferating subset in immunized mice followed by B lymphocytes, at least at this time point post immunization (**Figures 5C, D, G, H**). Altogether these results indicate that CD4^+^ lymphocytes from immunized mice showed a higher proliferation activity after incubation with H7-stimulated BMDC than control mice. In addition, no significant proliferation was observed after incubation of splenocytes or LN leukocytes with non-stimulated BMDC in immunized or non-immunized mice, supporting the specificity of the proliferative response carried out by committed lymphocytes during immunization.

#### 3.3.2 Cytokine response

Profile of cell-mediated immunity was evaluated by measuring cytokines (IL-4, IL-5, IL-17A and IFN-γ) in culture supernatants from H7-stimulated or unstimulated BMDC cultured with LN leukocytes and splenocytes (**Figures 5I, J**). Culture supernatants from LN and spleen cell suspensions from immunized mice incubated with H7-stimulated BMDC showed statistically significant higher IL-4, IL-5, IL-17A and IFN-γ concentrations compared to cultures with non-stimulated BMDC, supporting the H7-specifc response after immunization. In contrast, similar cytokines concentrations were produced by lymphocytes from control mice cultured with H7-stimulated and non-stimulated BMDC. Thus, significantly higher concentrations of all cytokines were observed in cultures from LN leukocytes and splenocytes with H7-stimulated BMDC from immunized mice compared to controls.

### 3.4 *In vivo* studies

#### 3.4.1 Delayed type of hypersensitivity

**Figure 6:**
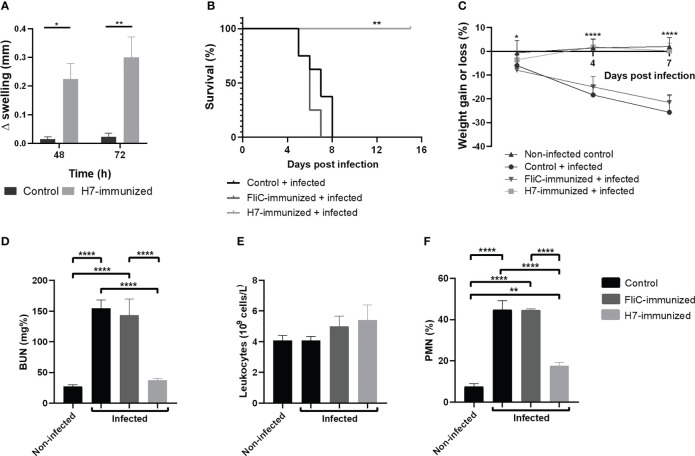
*In vivo* studies. **(A)** Delayed type hypersensitivity (DTH) assay on day 83 post first dose. Difference in footpad swelling (Δmm swelling) was measured in control and immunized mice at 48 and 72 h post H7- inoculation as described in Materials and Methods. Each bar represents the mean ± SEM of 4 mice per group. This experiment is representative of three biological replicates. Data was analyzed by two-way ANOVA test with Tukey’s post-test. **(B–F)** Challenge with 125/99 pW strain. Control (n=8), FliC and H7-immunized mice (n=8-9) were intragastrically infected with 10^5^ CFU of 125/99 pW on day 41 post first dose, as detailed in Materials and Methods. Daily after infection, mice were weighed and on day 7 p.i., total and differential leukocyte counts in whole blood and plasma BUN levels were measured. **(B)** Survival rates. Data was analyzed by Log Rank-test. **(C)** Percentage of weight loss or gain. Data was analyzed by two-way ANOVA test with Tukey’s post-test. **(D)** BUN levels; **(E)** Total leukocyte count; **(F)** Polymorphonuclear (PMN) count. Data was analyzed by one-way ANOVA test with Tukey’s post-test. *p<0.05, **p<0.01, ****p<0.0001.

The specific cellular response was evaluated *in vivo* by DTH reaction at day 83 after the first immunization dose. The inflammatory reaction in the footpad was measured in immunized and control mice after H7 injection. As depicted in **Figure 6A**, specific swelling was significantly higher in immunized mice that in controls at 48 and 72 h. This result indicates that a memory T cell systemic response has been generated by H7 nasal immunization.

#### 3.4.2 *Escherichia coli* O157:H7 challenge

To evaluate the protection against systemic disease secondary to *E. coli* O157:H7 infection after H7-immunization, immunized and control groups were intragastrically challenged with a lethal dose of the pathogenic bacteria (125/99 pW strain) on day 41 post first immunization dose. All non-immunized and FliC-immunized mice gradually lost weight and died one week after infection, showing high levels of BUN in plasma and a significant neutrophilia, compared to H7-immunized mice. In sharp contrast, all H7-immunized mice survived, maintaining their body weight and healthy status, normal levels of plasmatic BUN and percentage of neutrophils (% PMN) (**Figures 6B–F**). Thus, H7-immunized mice did not show clinical evidence of systemic disease after the same *E. coli* O157:H7 challenge that led control or immunized mice with unrelated flagella (FliC) to die.

#### 3.4.3 *Escherichia coli* O157:H7 intestinal colonization and barrier function after challenge

**Figure 7:**
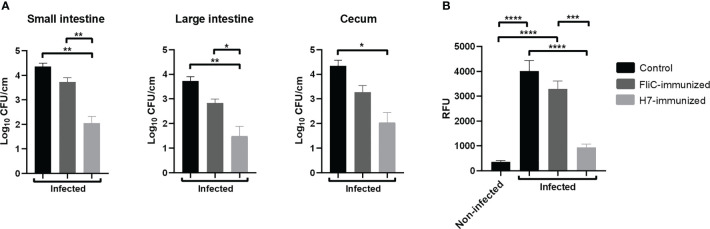
Colonization and intestinal barrier function after *E. coli* O157:H7 infection. H7-immunized (n=5), FliC-immunized (n=4) and non-immunized (n=3) mice were euthanized at 6 days p.i. to determine colonization in the small intestine, large intestine, and cecum. **(A)** The graphs represent the CFU of O157:H7/cm of each intestinal segment from infected mice. **(B)** Intestinal permeability. All infected mice and the control non-infected received orally 0.1 mL PBS containing FITC-Dx at 6 days p.i. After 4 h of treatment, they were bled to determine relative fluorescence units (RFU) in plasma. Each bar shows the mean ± SEM of 3-5 mice for each group. The data was analyzed by one-way ANOVA test with Tukey’s post-test: *p < 0.05; **p < 0.01; *** p < 0.001; ****p < 0.0001.

As *E. coli* O157:H7 colonization is the first step in the pathogenesis of HUS and flagella participate in the adherence of bacteria to the intestinal cells, we analyzed the level of intestinal colonization in H7- or FliC-immunized and non-immunized mice. The number of O157:H7 CFU recovered from small and large intestines, and cecum of mice at 6 days post challenge was quantified. H7-immunized mice showed a significant lower number of CFU/cm recovered from the three intestinal segments compared to non-immunized mice, as well as a significant lower colonization in small and large intestines compared to FliC-immunized mice (**Figure 7A**). In contrast, FliC-immunized mice did not show statistically significant differences in the level of colonization compared with non-immunized mice in any of the intestinal segments.

In addition, functional damage of intestinal barrier was evaluated by using the FITC-Dx assay in all experimental groups at 6 days post challenge. FliC-immunized and control infected mice showed a higher level of FITC-Dx in plasma compared to H7-immunized or non-infected mice (**Figure 7B**). In addition, H7-immunized infected mice did not show significant differences in the level of FITC-Dx in plasma compared to non-infected control mice. These results altogether, clearly demonstrate that anti-H7 mucosal antibodies are effective to reduce intestinal colonization and to prevent epithelial damage, leading to protection against the systemic disease secondary to gastrointestinal *E. coli* O157:H7 infection.

#### 3.4.4 Biochemical parameters and immune response against *Escherichia coli* O157:H7 in immunized surviving mice

**Figure 8:**
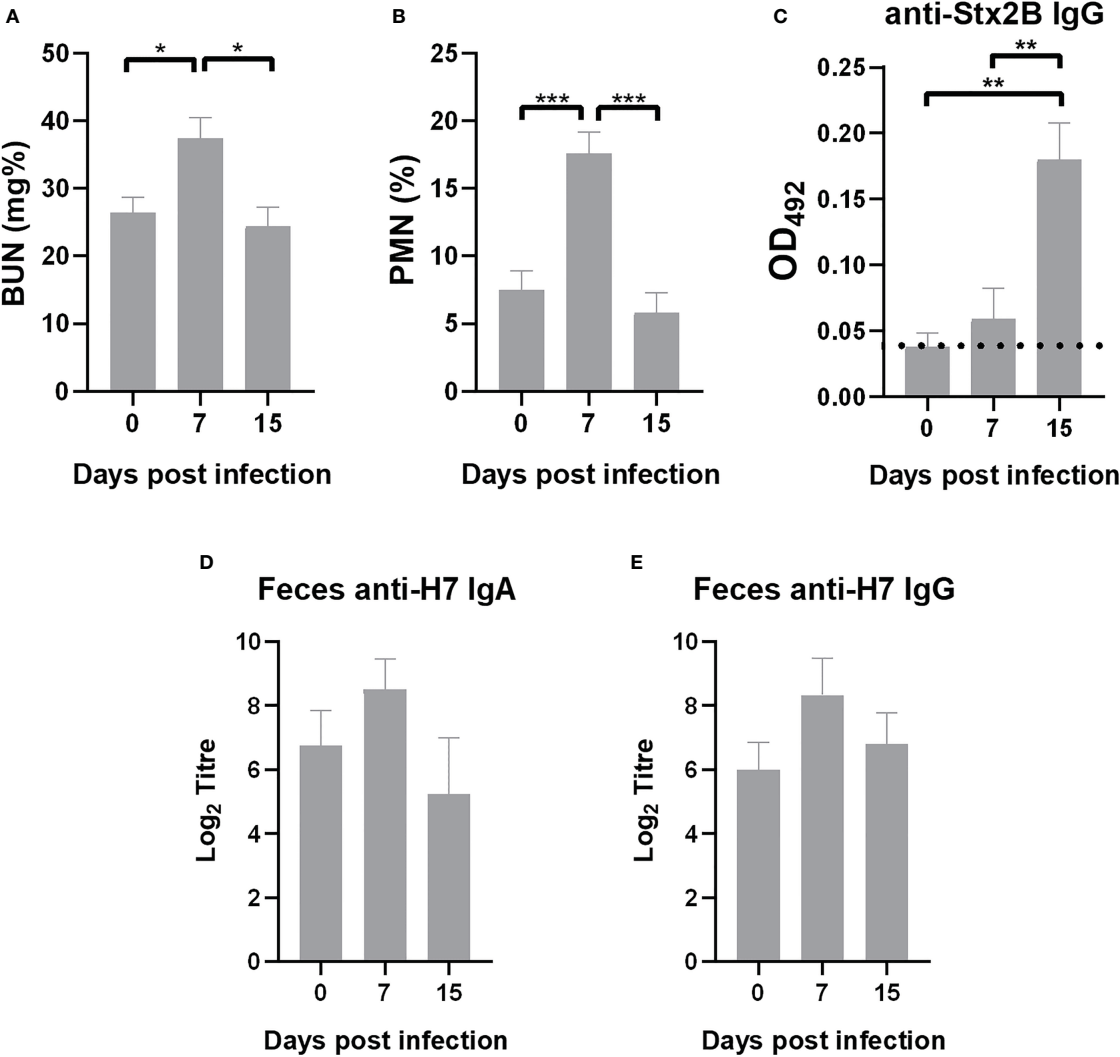
Outcome of immunized mice after infection. Surviving mice were bled at days 7 and 15 p.i. Data of biochemical parameters was compared with values previously to infection (day 0). **(A)** BUN levels. **(B)** PMN count. **(C)** Anti-Stx2B specific IgG levels measured in plasma (1/10 dilution) by ELISA expressed as OD492. Dotted line indicates anti-Stx2B IgG level in non-immunized mice. **(D, E)** Anti-H7 IgA and IgG in fecal supernatants measured by ELISA. Each bar represents the antibody titre mean ± SEM of 9 mice per treatment at the time indicated, expressed as the base 2 logarithm (Log_2_). The antibody titre corresponds to the last dilution of the fecal supernatant that is statistically significant compared to fecal supernatants of control mice (non-immunized non-infected mice). Data was analyzed by one-way ANOVA test with Tukey’s post-test. *p<0.05, **p<0.01, ***p<0.001.

Health status and immune response was evaluated in surviving immunized mice after 7 and 15 days of the lethal challenge with *E. coli* O157:H7. BUN values and %PMN returned to baseline values at 15 days (**Figures 8A, B**). Humoral immune response against pathogenic factors from bacterial strain were also evaluated. Surviving immunized mice presented a significant increase in plasmatic level of anti-Stx2 antibodies at 15 days compared to 0 and 7 days p.i. (**Figure 8C**), indicating that *E. coli* O157:H7 infection elicited a specific protective response in immunized mice. In addition, levels of anti-H7 IgG and IgA in fecal supernatants from immunized mice after infection were similar to those observed before infection, suggesting that these antibodies were not depleted by bacterial opsonization during the course of *E. coli* O157:H7 infection (**Figures 8D, E**).

## 4 Discussion

In the present study we showed that intranasal immunization of BALB/c mice with H7 flagellin successfully induced a specific and protective immunity against the lethal systemic disease secondary to *E. coli* O157:H7 gastrointestinal infection. Among previous approaches directed to block the gastrointestinal phase of the disease in humans, Ahmed et al. (2006) developed polysaccharide conjugate vaccines based on detoxified LPS from *E. coli* O157. Phase I and phase II clinical studies were carried out in adults and children. The *E. coli* O157 conjugate vaccines were safe for all ages, and a positive humoral IgG response with bactericidal activity was found in serum from both age vaccinated populations. However, there were certain limitations for using LPS-based vaccines. Among them, LPS failed to induce a long-lasting humoral immune response especially in children, and the intestinal humoral response was not evaluated (Ahmed et al., 2006).

Although the efficacy of vaccines is the most important issue, the action mechanism in terms of host immune responses is also desirable to be elucidated in order to take forward a step in vaccine development.

Having established that no other pathogenic factors were detected in H7 flagellin batches, we analyzed the specific immune responses *in vitro* and *in vivo*. In the present work, we have shown that H7 flagellin mucosal immunization without any additional adjuvant was enough to trigger antigen-specific T and B cell responses in mice.

Activation of DC by bacterial components *via* TLR is a major event during the induction of immune responses. In this regard, we demonstrated that H7 was able to activate CD11c^+^ BMDC *in vitro* evidenced by the induction of CD86 surface expression. The expression of the co-stimulatory CD86 antigen is essential to guarantee T cell-specific responses. In addition, H7-stimulated BMDC secreted significant levels of IL-6 and IL-12 compared to unstimulated BMDC, but lower levels compared to LPS-stimulated BMDC, and not significant levels of TNF-α. Although different profiles of cytokine secretion by BMDC stimulated *in vitro* have been reported according to the mouse strain or culture conditions (concentration of antigen, time of incubation, etc.), our results agree with previous reports (Bates et al., 2009). One hypothesis that explains the lower DC response to flagellin compared to other TLR agonists is that only a small fraction of the total DC population express TLR5. However, flagellin from gram-negative bacteria have an extremely high affinity for these receptors (Mizel et al., 2003), resulting in a very efficient uptake of flagellin by DC through a TLR5 dependent endocytosis. This effect could explain the enhanced antigen-specific immune response *in vivo*. The apparent lower capacity to activate DC compared to LPS could represent an advantage in the mucosal epithelia, considering that a strong inflammatory response could have deleterious effects. In addition, H7 also induced IL-12 p70 secretion by BMDC, which is a major Th1-polarizing cytokine (Bates et al., 2009). Anyway, it has been reported that flagellin *in vivo* activity would result from the synergy of three distinct processes: direct activation of TLR5 DC, cytokine and chemokine production by non-DC, and activation of the vascular endothelium (Bates et al., 2009).

Lymphocytes, particularly the CD4^+^ subpopulation from the lung-draining LN and spleens of immunized mice showed a high proliferative activity in response to H7-stimulated BMDC. To evaluate Th1/Th2/Th17 profile of cell-mediated immune response elicited in immunized mice, IL-4, IL-5, IL-17A and IFN-γ concentrations were measured in culture supernatants of antigen-specific lymphocyte from immunized mice after 48 days of the first immunization dose. We found that all these cytokines were produced by lymphocytes from the draining LN and spleen of immunized mice in response to H7-stimulated BMDC. Th2-related cytokines (IL-4 and IL-5) are known to activate B-cell differentiation and terminal maturation of IgG and IgA *producing B cells*, and support the generation of long-lasting memory B cells (Granato et al., 2014; Hashiguchi et al., 2020). IL-17 is known to induce an innate-like acute immune response in mucosal tissue through secretion of chemokines (including IL-8), IL-6 and G-CSF, which attract myeloid cells, together with antimicrobial peptides production (McGeachy et al., 2019), thus enhancing the barrier function (Mills, 2022). In this sense, the induction of Th17 cells after H7-immunization would contribute to protect the host during acute microbial infection. These results are consistent with previous findings that demonstrated that flagellin induces the production of not only IFN-γ, but also IL-4 and IL-13 by lymphocytes *in vivo*, and this Th2-biased differentiation is associated with MyD88-dependent activation of DC (Didierlaurent et al., 2004; Vijayan et al., 2018).

In this work we observed DC and lymphocyte activation, which is central for sustaining the effector and regulatory arms of specific immune responses as well as long-lasting memory response. Most importantly, we observed that nasal immunization with H7 flagellin led to a significant rise of anti-H7 antibodies at both systemic and intestinal mucosa level. These results are in agreement with other reports demonstrating that immunization at one mucosal surface induces an antigen-specific antibody response at distal mucosal surfaces. In fact, H7 flagellin was able to evoke IgA responses in the intestinal mucosa (Asadi Karam et al., 2016). Regarding the finding of IgG in fecal supernatants, it could be the result of passive passage from systemic circulation as well as local production. A similar anti-FliC specific humoral immune response was observed in FliC-immunized mice, indicating that flagellar antigens trigger a strong immune response. In addition, plasma and fecal samples from these mice specifically react with FliC but not with H7 antigens in ELISA assays.

In addition, we demonstrated H7-specific memory T cells *in vivo* after 83 days from the beginning of the immunization scheme. In fact, DTH is a local inflammatory reaction mediated by CCR7- effector memory T lymphocytes at the site of injection of the antigen against which the immune system has been primed (Beeton and Chandy, 2007). Thus, the significant increase in swelling observed in the footpads from immunized mice compared to controls confirmed that H7 triggered an efficient T memory response *in vivo.*

All these immunologic mechanisms are probably involved in conferring specific protection to mice against a lethal dose of the virulent *E. coli* O157:H7. In fact, whereas FliC-immunized and non-immunized mice died as a consequence of the Stx2-induced kidney injury, H7-immunized mice showed only a slight increase in BUN concentration and peripheral neutrophils that restored to baseline at 15 days p.i. Moreover, H7-immunized mice showed a significantly lower level of intestinal colonization than non-immunized or FliC-immunized mice. Besides, FliC-immunized and non-immunized mice showed an increased permeability through the intestinal barrier after the *E. coli* O157:H7 challenge compared to H7-immunized mice. In this sense, the increased permeability is probably the consequence of the higher intestinal colonization, which may induce a higher intestinal inflammation and damage and decreased barrier function in control groups compared to H7- immunized mice.

A remarkable finding was the presence of specific anti-Stx2B IgG in plasma as well as anti-H7 antibodies in fecal supernatants from infected and immunized mice at 15 days after challenge. This data suggests that during STEC infection the immune response is stimulated, leading to the induction of circulating anti-Stx2 antibodies, and because local antibodies were not totally consumed, bacterial challenge acted probably as a booster stimulus. This observation is very important for Argentina, where there is a high circulation of pathogenic STEC strains and it would be desirable that vaccination does not prevent immune responses against other virulence factors during subsequent gastrointestinal infections.

## Data availability statement

The raw data supporting the conclusions of this article will be made available by the authors, without undue reservation.

## Ethics statement

The Institutional Animal Care and Use Committee at IMEX CONICET-Academia Nacional de Medicina approved all procedures in accordance with the principles set forth in the Guide for the Care and Use of Laboratory Animals (National Research Council (US) Committee, 2011), (protocol number 86/2021).

## Author contributions

MP and MRu contributed to the conception and design of the article. AB, FS and MT contributed to experimental development, and interpretation of data. DM, MV and AE performed some experiments. RF-B and MVR contributed to analysis of results and the statistical analysis. All authors contributed to the article and approved the submitted version.
