# Supplementary material for: Nasal immunization with H7 flagellin protects mice against hemolytic uremic syndrome secondary to Escherichia coli O157:H7 gastrointestinal infection
Source: Front Cell Infect Microbiol. 2023 May 16;13:1143918. doi: 10.3389/fcimb.2023.1143918 (PMC10227447; doi:10.3389/fcimb.2023.1143918)
Supplement: Supplementary file 1 [file DataSheet_1.docx]

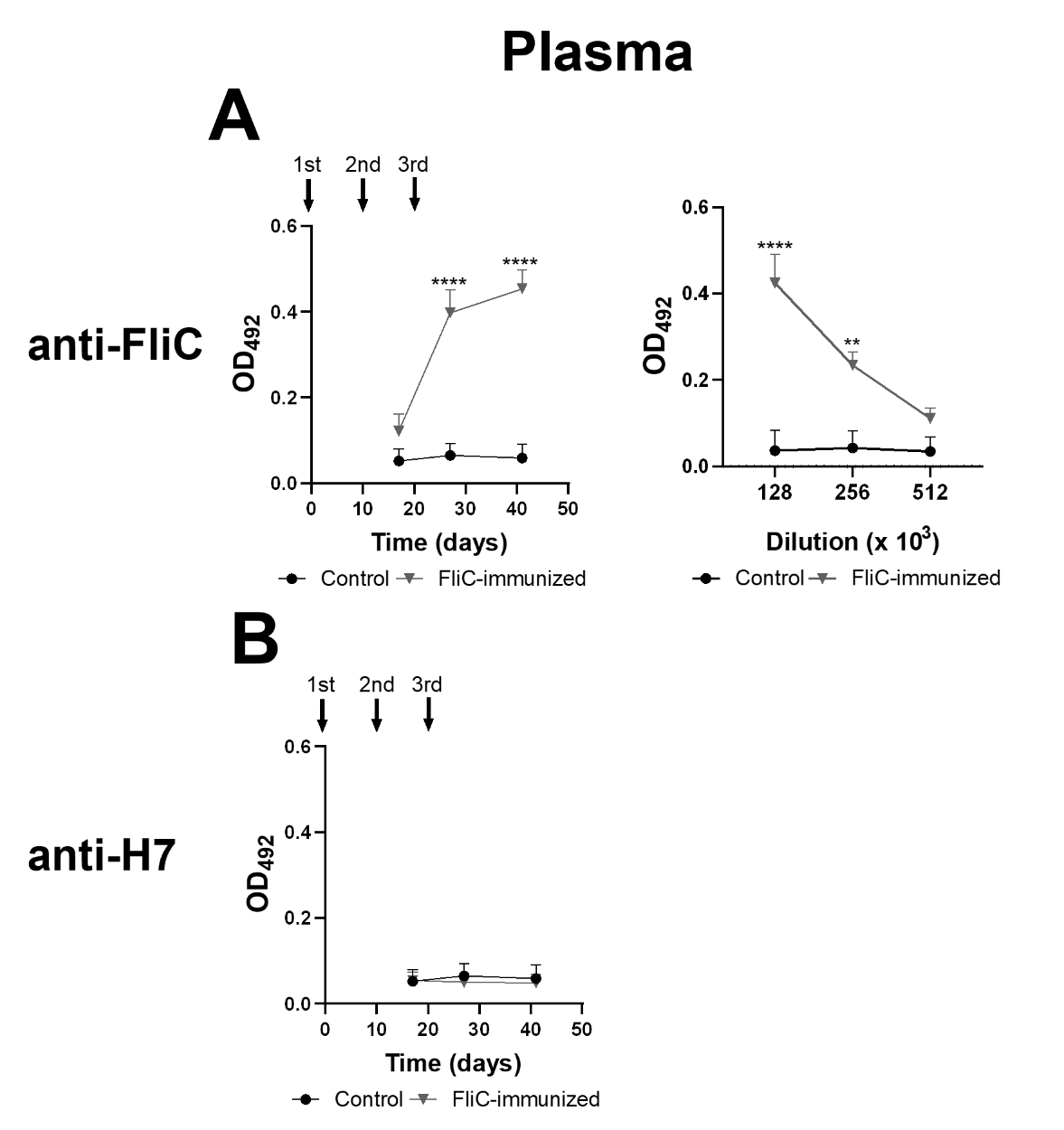


**Supplementary Figure 1. Levels of anti-FliC and anti-H7 IgG measured by ELISA in plasma. A) Left graph:** The solid arrows indicate the timing of immunizations with FliC. Each point shows anti-FliC IgG level in plasma at 1/128000 dilution expressed as OD_492_ at each time point. **Right graph:** Antibody titre at the peak of maximum response post immunization (day 41). **B)** Each point shows anti-H7 IgG level in plasma at 1/32000 dilution expressed as OD_492_ at each time point. Each point represents the mean ± SEM of 4 control and immunized mice. Data was analyzed by two-way ANOVA test with Tukey’s post-test. **p<0.01, ****p<0.0001.


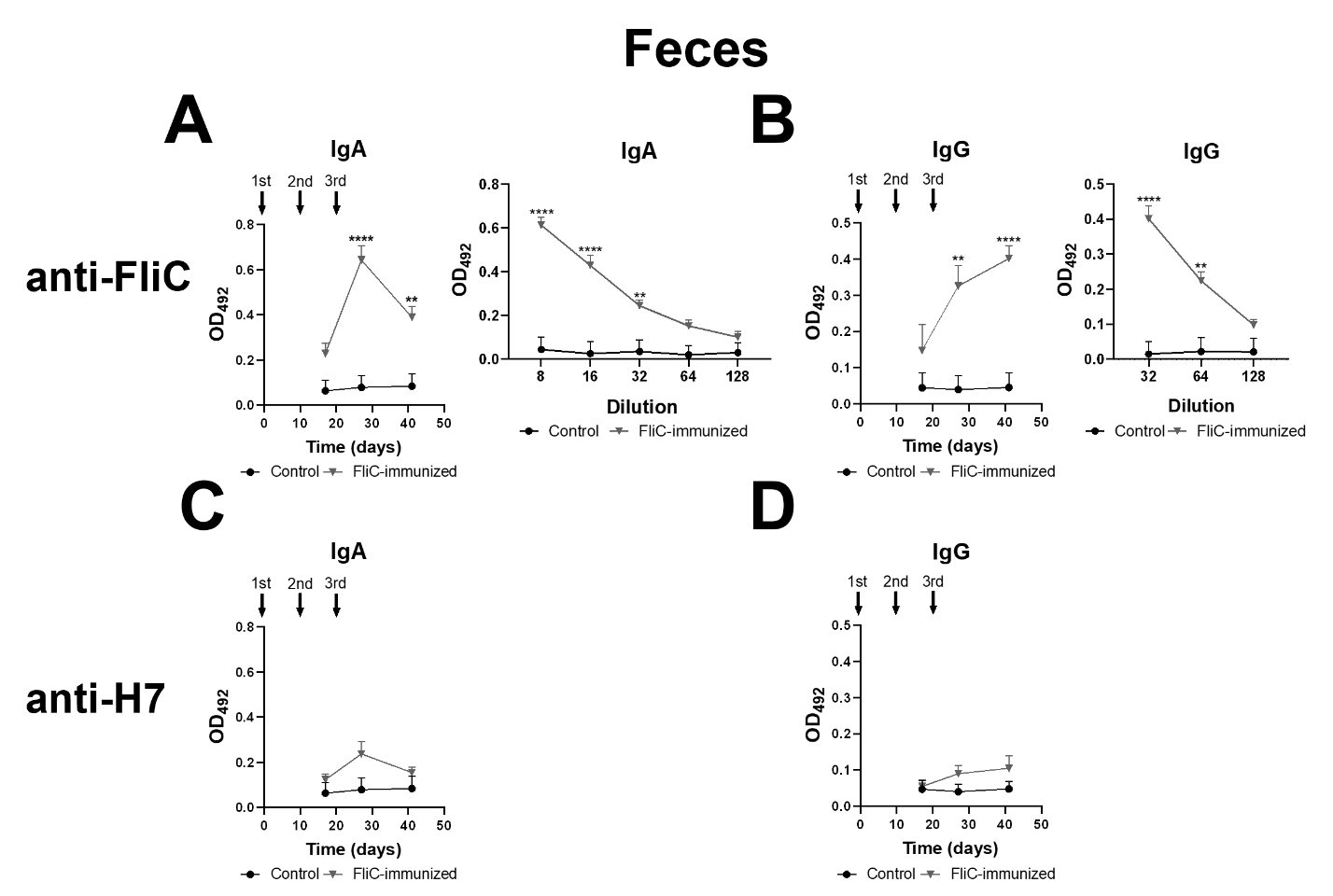


**Supplementary Figure 2. Levels of anti-FliC and anti-H7 IgG and IgA measured by ELISA in feces.** The solid arrows indicate the timing of immunizations with FliC. **A) Left graph:** Each point shows anti-FliC IgA levels in fecal supernatants at 1/8 dilution, expressed as OD_492_ at each time point. **Right graph:** Anti-FliC IgA titres at the peak of maximum response post immunization (day 27). **B) Left graph:** Each point shows anti-FliC IgG levels in fecal supernatants at 1/32 dilution, expressed as OD_492_ at each time point_._ **Right graph:** Anti-FliC IgG titres at the peak of maximum response post immunization (day 41). **C and D)** Each point shows anti-H7 IgA and IgG levels in fecal samples at 1/8 and 1/32 dilution, respectively, expressed as OD_492_ at each time point. Each point shows the mean ± SEM of 4 control and immunized mice. Data was analyzed by two-way ANOVA test with Tukey’s post-test. **p<0.01, ****p<0.0001.
